# The *Lactococcus lactis* KF147 nonribosomal peptide synthetase/polyketide synthase system confers resistance to oxidative stress during growth on plant leaf tissue lysate

**DOI:** 10.1002/mbo3.531

**Published:** 2017-09-18

**Authors:** Benjamin L. Golomb, Annabelle O. Yu, Laurynne C. Coates, Maria L. Marco

**Affiliations:** ^1^ Department of Food Science and Technology University of California Davis CA USA; ^2^ Present address: Bayer U.S. LLC Crop Science Division West Sacramento CA USA

**Keywords:** *Lactococcus lactis*, natural products, NRPS/PKS, Plant‐associated bacteria, reactive oxygen species, secondary metabolites

## Abstract

Strains of *Lactococcus lactis* isolated from plant tissues possess adaptations that support their survival and growth in plant‐associated microbial habitats. We previously demonstrated that genes coding for a hybrid nonribosomal peptide synthetase/polyketide synthase (NRPS/PKS) system involved in production of an uncharacterized secondary metabolite are specifically induced in *L. lactis* KF147 during growth on plant tissues. Notably, this NRPS/PKS has only been identified in plant‐isolated strains of *L. lactis*. Here, we show that the *L. lactis* KF147 NRPS/PKS genes have homologs in certain *Streptococcus mutans* isolates and the genetic organization of the NRPS/PKS locus is conserved among *L. lactis* strains. Using an *L. lactis* KF147 mutant deficient in synthesis of NrpC, a 4′‐phosphopantetheinyl transferase, we found that the NRPS/PKS system improves *L. lactis* during growth under oxidative conditions in *Arapidopsis thaliana* leaf lysate. The NRPS/PKS system also improves tolerance of *L. lactis* to reactive oxygen species and specifically H_2_O_2_ and superoxide radicals in culture medium. These findings indicate that this secondary metabolite provides a novel mechanism for reactive oxygen species detoxification not previously known for this species.

## 1 INTRODUCTION

Numerous bacterial species in multiple phyla possess the capacity to synthesize secondary metabolite peptides and carboxy acids by nonribosomal peptide synthetases (NRPS) and polyketide synthases (PKS) (Weissman, 2015). These secondary metabolites can include proteinogenic and nonproteinogenic amino acids and ketides assembled into diverse end‐products with functions ranging from antibiotics to immunosuppressants (Siezen & Khayatt, 2008). NRPS/PKS systems have been described in detail for many industrially significant bacteria but there are few reports characterizing these systems and their resulting products by lactic acid bacteria (LAB) (Lin et al., 2015; Wu et al., 2010).

LAB are gram‐positive, nonsporulating bacteria in the *Firmicutes* phylum and constitute a genetically diverse collection of species characterized by their fermentation of mono‐ and di‐saccharides to lactic acid. Strains of *Lactococcus lactis* are among the most extensively characterized LAB and are used in a variety of applications including starter cultures in cheese production (Cavanagh, Fitzgerald, & McAuliffe, 2015), industrial product synthesis (Mierau et al., 2005), and for the delivery of therapeutics to the human gastrointestinal tract (Cano‐Garrido, Seras‐Franzoso, & Garcia‐Fruitos, 2015; Wells & Mercenier, 2008). Although *L. lactis* has been most extensively investigated for its adaptation to dairy environments, it is generally regarded that plants are the ancestral habitat for this species (Cavanagh, Fitzgerald, & McAuliffe, 2015; Cavanagh, Casey et al., 2015). *L. lactis* KF147, a strain originally isolated from mung bean sprouts, is genetically distinct from dairy‐associated isolates (Siezen et al., 2008). Its unique traits include the ability to metabolize a wide array of plant‐derived carbohydrates and synthesize exopolysacharides (Siezen et al., 2008).

The genome of *L. lactis* subspecies *lactis* KF147 contains a hybrid NRPS/PKS gene cluster (Siezen et al., 2008, 2010). These chromosomally‐located genes have thus far only been identified in plant‐derived lactococci (Siezen et al., 2011). However, the conditions in which those genes are expressed were not described. Because the production of secondary metabolites poses a significant energy burden on the cell, genes necessary for the production of those cell products are frequently cryptic in standard laboratory media (Rutledge & Challis, 2015). In fact, stringent transcriptional control and regulation of NRPS/PKS production is a frequent challenge that impedes the study of these systems and other secondary metabolites (Rutledge & Challis, 2015). Therefore, it was notable when we found that the *L. lactis* KF147 genes coding for the hybrid NRPS/PKS are induced during the growth of this strain in *Arabidopsis thaliana* leaf tissues (Golomb & Marco, 2015). The findings provided an opportunity to study the functional relevance of the NRPS/PKS system in *L. lactis*. Here, we examine the genetic relatedness of the NRPS/PKS to other lactococci and *Streptococcus mutans* strains and demonstrate the contribution of the secondary metabolite to *L. lactis* growth in oxidative conditions, most appreciably on plant tissues.

## 2 EXPERIMENTAL PROCEDURES

### 2.1 Bacterial strains and culture conditions

*Lactococcus lactis* subsp. *lactis* KF147 (Kelly, Davey, & Ward, 1998) was provided by NIZO food research (Ede, The Netherlands) and was maintained as a frozen glycerol stock at −80°C. *L. lactis* was routinely grown at 30°C without agitation on M17 broth (BD, Franklin Lakes, NJ) supplemented with 0.5% d‐glucose (GM17). *Arabidopsis thaliana* Col‐1 leaf lysate (ATL medium) was prepared as previously described (Golomb & Marco, 2015). *Escherichia coli* DH5α was grown at 37°C on LB (Fisher Scientific, Waltham, MA). When necessary, the culture medium was supplemented with ampicillin at 100 μg/ml for *E. coli* and erythromycin at 5 μg/ml for *L. lactis*. For reactive oxygen species tolerance experiments, optical density (OD) at 600 nm was used to examine *L. lactis* growth in GM17 in a Synergy 2 microplate reader (BioTek, Winooski, VT) in 200 μl volumes. *L. lactis* numbers were also determined by plating serial dilutions of the cultures onto GM17 agar and incubation at 24 hr prior to viable cell enumerations. *L. lactis* used for growth experiments was prepared from stationary phase cells incubated in GM17 for approximately 18 hr. Cells were collected by centrifugation (10,000*g*, 3 min) and washed twice in phosphate‐buffered saline [PBS, 137 mmol/L NaCl, 2.7 mmol/L KCl, 4.3 mmol/L Na_2_HPO_4_, 1.4 mmol/L KH_2_PO_4_ (pH 7)] prior to use.

### 2.2 Sequence analysis of the *L. lactis* KF147 NRPS/PKS genes

Annotated NRPS/PKS genes were retrieved from the complete genome sequence of KF147 (Siezen et al., 2010). Homology searches were performed, using BLASTp against the NCBI nr database (http://blast.ncbi.nlm.nih.gov/Blast.cgi).

### 2.3 Construction of the *L. lactis nrpC* deletion mutant BAL1

Standard molecular biology techniques were performed as previously described (Sambrook, Fritsch, & Maniatis, 1989). Double‐crossover homologous recombination was used to generate a markerless *nrpC* deletion mutant of KF147, using the suicide vector pRV300 (Leloup, Ehrlich, Zagorec, & Morel‐Deville, 1997). All primers used for mutant construction are shown in Table S1. The upstream flanking region of *nrpC* was PCR amplified, using primers A and B and the downstream flanking region was PCR amplified, using primers C and D. The resulting amplicons were combined by splicing‐by‐overlap extension (SOEing) PCR (Horton, Cai, Ho, & Pease, 1990). The resulting PCR product was digested with *Sal*I and *Sac*II, cloned into pRV300, and transformed into *E. coli* DH5α to yield pNRPC‐KO. pNRPC‐KO was next introduced into KF147 by electroporation as previously described (Golomb & Marco, 2015). Single‐crossover mutants were selected on GM17 agar plates supplemented with erythromycin. A single‐crossover mutant grown on GM17 broth in the absence of erythromycin for approximately eight passages was sufficient to facilitate excision of the plasmid. Double‐crossover mutants were identified by replica plating onto GM17 agar supplemented with erythromycin. Erythromycin‐sensitive colonies were screened by PCR for a *nrpC* deletion. PCR products with primers A and D that resulted in a product size of 1125 bp indicated the absence of *nrpC* and pRV300 from the chromosome (Table S1). The PCR products were also sequenced (UC Davis DNA Sequencing Facility (http://dnaseq.ucdavis.edu) for confirmation. A single *nrpC* deletion mutant, strain BAL1, was used in subsequent experiments.

### 2.4 ROS tolerance

The capacity of *L. lactis* to tolerate Reactive oxygen species (ROS) was examined in multiple ways including (1) growth in H_2_O_2,_ (2) paraquat (methyl viologen dichloride hydrate), (3) xanthine with xanthine oxidase (XO), and (4) under respiratory conditions in the presence of hemin. For these studies, GM17 was supplemented to a final concentration with either 0.5 mmol/L H_2_O_2_ (Macron Fine Chemicals, Center Valley, PA), 40 mmol/L paraquat (Sigma‐Aldrich, St. Louis, MO), or 5 mmol/L xanthine (Sigma‐Aldrich) in the presence of 0.3 U/ml XO (EMD Millipore, Billerica, MA). For the growth in the presence of xanthine, controls were supplemented with xanthine but not XO. In ATL, 10 mmol/L paraquat was used. For growth under respiratory conditions, *L. lactis* was incubated with continuous aeration in GM17 supplemented with porcine hemin at a final concentration of 10 μg/ml (Chem‐Impex International, Wood Dale, IL) and where indicated, 20 mmol/L paraquat.

### 2.5 Statistical analysis

Significant differences in *L. lactis* growth rates were determined using an unpaired, two‐tailed Student t‐test. Differences were considered significant if *p *<* *.05.

## 3 RESULTS AND DISCUSSION

### 3.1 The NRPS/PKS locus contains distinct functional features and is conserved among plant‐associated *L. lactis* strains

**Figure 1:**
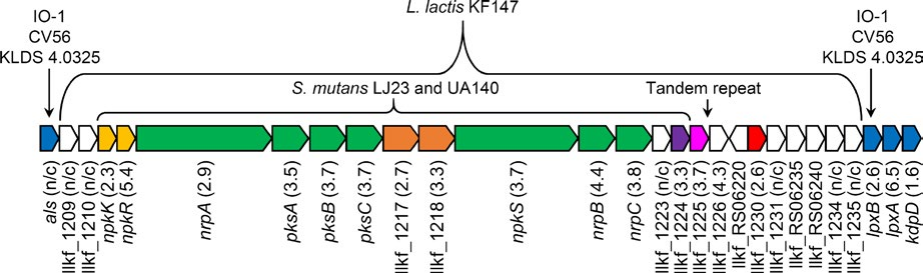
The hybrid NRPS/PKS from *L. lactis* KF147. Genes on the genomic island are colored as follows: two‐component transcriptional regulator (yellow), secondary metabolite biosynthesis enzymes (green), ABC transporter (orange), macrolide biosynthetic protein AvrD (purple), phytoene dehydrogenase (pink), DNA integration/recombination/inversion protein (red), and hypothetical proteins (white). Insertion of the genomic island is shown relative to *L. lactis* strains originating from different sources (strains IO‐1, CV56, and KLDS 4.0325) genes in blue. The fold‐induction of each gene in ATL relative to GM17 is shown in parentheses as determined previously (Golomb & Marco, 2015). N/C indicates no change in expression was observed. Gene expression results were not reported for llkf_RS06220, llkf_RS06235, and llkf_RS06240 because they are newly annotated open reading frames

The *L. lactis* KF147 genes coding for the NRPS/PKS system (llkf_1211 to llkf_1222) span over 40 kb (ca. 1.5% of the chromosome) and consist of a two‐component response regulator (*npkKR*), biosynthetic enzymes (*nrpA*,* pksABC*,* npkS*, and *nrpB*), a 4′‐phosphopantetheinyl transferase (PPTase, *nrpC*), and a putative ATP‐binding cassette (ABC) transporter (llkf_1217 and llkf_1218) (Figure 1). The complete locus spans from llkf_1209 to llkf_1235 and includes the two accessory genes llkf_1224 annotated as an AvrD macrolide biosynthetic protein and llkf_1125 annotated as a phytoene dehydrogenase (Figure 1). Finally, there are multiple hypothetical proteins (Figure 1).

Flanking one end of the NRPS/PKS locus in *L. lactis* KF147 is a DNA integration/recombination/inversion protein (llkf_1230) (Figure 1). This protein is similar to lactococcal and streptococcal phage integrases (30%–93% amino acid similarity) and shares a C‐terminal catalytic domain with the *Bacillus subtilis* ICE*Bs1* conjugative transposon integrase. The integrase is required in *B. subtilis* for ICE*Bs1* excision and integration (Lee, Auchtung, Monson, & Grossman, 2007). Another feature of the ICE*Bs1* transposon is the formation of repeated nucleotide sequences (Wozniak & Waldor, 2010). This is similar to the action of *L. lactis* Tn*6098* (Machielsen, Siezen, van Hijum, & Vlieg, 2011). Using Tandem Repeat Finder (Benson, 1999), we identified a 21 bp repeat (ACTATAATAAAAGATGGAGTG) between llkf_1225 and llkf_1226 in KF147 (and NCDO 2118), which might have been the insertion site for the NRPS/PKS (Figure 1). Notably, the surrounding hypothetical proteins do not share homology with other ICE*Bs1* genes and the G+C contents of the genes in the locus (35.3%) do not differ from the chromosome (34.9%). Therefore, these genes may have been acquired by horizontal gene transfer from a related bacterial species and constitute a genomic island. Alternatively, the NRPS/PKS system and flanking genes might have been present in an ancestral lactococcal genome and subsequently lost during adaptation to new habitats such as milk.

The same NRPS/PKS system is also present in the published genomes of *L. lactis* strains NCDO 2118 (completed, isolated from frozen peas) (Oliveira et al., 2014) and YF11 (draft, isolated from fermented corn) (Cavanagh, Fitzgerald, & McAuliffe, 2015; Du et al., 2013). Genes coding for the NRPS/PKS system were also detected in *L. lactis* Li‐1, KF134, KF146, and KF196 according to comparative genome hybridization (Siezen et al., 2011) and confirmed by BLASTp analysis (data not shown). Each of these strains was isolated from (fermented) plant foods and are classified to the *L. lactis* subspecies *lactis*. Due to the high sequence conservation (>99%) amongst the NRPS/PKS systems in these strains, we expect them to synthesize the same secondary metabolite.

Inspection of the genes flanking the NRPS/PKS locus in *L. lactis* strains KF147 and NCDO2118 showed that they are inserted relative to other *L. lactis* strains between genes encoding acetolactate synthase (*als*, upstream) and two hypothetical LPxTG membrane proteins (*lpxAB*) followed by the *kdpDEABC* potassium transport system (downstream) (Figure 1 and Table 1). *L. lactis* strains that lack the NRPS/PKS system but share the same flanking gene arrangement as KF147 were isolated from other sources including strain IO‐1 (a kitchen sink isolate) (Kato et al., 2012), CV56 (a vaginal isolate) (Gao et al., 2011), and KLDS 4.0325 (a dairy isolate) (Yang, Wang, & Huo, 2013) (Figure 1). Importantly, even within the *lactis* subspecies, there is considerable variation in the NRPS/PKS flanking regions because not all strains display this genetic composition. In this regard, the dairy‐associated *L. lactis* strain IL1403 does not share the same gene arrangement. In IL1403, *als* is located on a distal part of the chromosome and this strain lacks *kdpDEABC*.

**Table 1:** BLASTp results of *L. lactis* KF147 NRPS/PKS genomic island^a^

*L. lactis* KF147				Top BLASTp hit			
Gene	Length (bp)	Accession	Annotation	Organism	Annotation	Similarity (%)	Coverage (%)
*als*	1665	WP_012897772.1	Acetolactate synthase	*L. lactis* b	Acetolactate synthase	99	100
llkf_1209	198	WP_012897773.1	Hypothetical protein	*Virgibacillus pantothenticus*	Hypothetical protein	53	92
llkf_1210	243	WP_012897774.1	Hypothetical protein	No hits			
*npkK*	1776	WP_012897775.1	Histidine kinase	*S. mutans* b ^*,*^ c	Histidine kinase	65	99
*npkR*	603	WP_012897776.1	DNA‐binding response regulator	*S. mutans* b ^*,*^ c	Two‐component system response regulator	78	100
*nrpA*	17136	WP_012897777.1	Nonribosomal peptide sythetase	*S. mutans* b ^*,*^ c	Nonribosomal peptide synthetase	69	99
*pksA*	1266	WP_012897778.1	PKS acyl‐CoA transferase	*S. mutans* b ^*,*^ c	PKS acyl‐CoA transferase	74	100
*pksB*	4533	WP_012897779.1	PKS biosynthesis protein	*S. mutans* b ^*,*^ c	PKS biosynthesis protein	68	99
*pksC*	4716	WP_012897780.1	PKS biosynthesis protein	*S. mutans* b ^*,*^ c	PKS biosynthesis protein	73	100
llkf_1217	861	WP_012897781.1	ABC transporter, ATP‐binding protein	*S. mutans* b ^*,*^ c	ABC transporter ATP‐binding protein	84	99
llkf_1218	738	WP_012897782.1	MFS transporter permease	*S. mutans* b ^*,*^ c	ABC transporter permease	78	100
*npkS*	6441	WP_012897784.1	polyketide synthase	*S. mutans* b ^*,*^ c	Polyketide synthase	67	99
*nrpB*	696	WP_012897785.1	Thioesterase	*S. mutans* b ^*,*^ c	Thioesterase	72	100
*nrpC*	669	WP_012897786.1	4′‐phosphopantetheinyl transferase	*S. mutans* b ^*,*^ c	4′‐phosphopantetheinyl transferase	70	100
llkf_1223	192	WP_012897787.1	Hypothetical protein	No hits			
llkf_1224	945	WP_012897788.1	Macrolide biosynthetic protein, AvrD family	*S. mutans* b ^*,*^ c	Macrolide biosynthetic protein, AvrD family	61	96
llkf_1225	1506	WP_012897789.1	Phytoene dehydrogenase	*Bacillus thuringiensis* b	Hypothetical protein	47	99
llkf_1226	651	WP_012897790.1	Hypothetical protein	*Enterococcus sp*.	ATP‐binding protein	38	95
llkf_RS06220	372	WP_012897763.1	Hypothetical protein	*Burkholderia pseudomallei*	Hypothetical protein	33	36
llkf_1230	1350	WP_012897791.1	DNA integration/ recombination/inversion protein	*Enterococcus durans*	Integrase	33	91
llkf_1231	1818	WP_012897765.1	Hypothetical protein	*Enterococcus faecalis* b	Hypothetical protein	34	97
LLKF_RS06235	1677	WP_012897795.1	Hypothetical protein	*Paenibacillus polymyxa* b	Hypothetical protein	31	98
llkf_RS06240	1851	WP_012897797.1	Hypothetical protein	*Desulfobulbus japonicus*	Hypothetical protein	32	96
llkf_1234	135	WP_012897767.1	Hypothetical protein	*L. lactis* b	Transposase	85	70
llkf_1235	936	WP_012897794.1	Hypothetical protein	*E. faecalis*	Hypothetical protein	55	91
*lpxB*	2229	WP_012897795.1	Hypothetical protein	*L. lactis* b	Hypothetical protein	76	96
*lpxA*	2469	WP_012897796.1	Hypothetical protein	*L. lactis* b	Hypothetical protein	99	100
*kdpD*	2631	WP_012897797.1	Two‐component system sensor histidine kinase KdpD	*L. lactis* b	Two‐component system sensor histidine kinase KdpD	99	100

a The same results were found for *L. lactis* strains NCDO 2118 and YF11.

b Multiple strains.

c Best hits are *S. mutans* LJ23 and UA140.

### 3.2 The *L. lactis* NRPS/PKS is homologous to *S. mutans* TnSmu2

Protein alignments of the *L. lactis* KF147 NRPS/PKS system revealed a high degree of amino acid similarity (65%–84%) and coverage (99%–100%) to an NRPS/PKS system in multiple strains of *Streptococcus mutans* on the TnSmu2 genomic island (Table 1) (Wu et al., 2010). The *L. lactis* KF147 NRPS/PKS system is most similar to *S. mutans* LJ23, a strain for which the complete genome sequence is available (Aikawa et al., 2012). None of the genes downstream of llkf_1224 had homologs in *S. mutans* LJ23, with the exception of the gene encoding the putative macrolide biosynthetic protein AvrD (llkf_1224). This gene is in the same location relative to the NRPS/PKS system in *S. mutans* LJ23 and shares 61% amino acid identity with 96% coverage (Table 1).

Although the function of the NRPS/PKS in *S. mutans* LJ23 has not been directly investigated, the same system in *S. mutans* UA140 was recently characterized (Wu et al., 2010). This NRPS/PKS product was the first to be characterized for a LAB species and shown to confer pigmentation and improve oxygen and hydrogen peroxide stress tolerance (Wu et al., 2010). The *S. mutans* UA140 NRPS/PKS locus is identical to that of *S. mutans* LJ23 (*personal communication*, Fengxia Qi) and therefore is also conserved with *L. lactis* KF147 (Figure 1 and Table 1).

### 3.3 ATL and paraquat in combination delay growth of the NRPS/PKS deletion mutant

We previously found significant increases in *L. lactis* KF147 NRPS/PKS gene transcript levels during growth in *Arabidopsis* leaf tissue lysate (ATL) compared to in GM17 (Golomb & Marco, 2015). Therefore, we investigated the importance of the NRPS/PKS system in conferring optimal growth rates to *L. lactis* in ATL by constructing strain BAL1, a markerless, *nrpC* deletion mutant of *L. lactis* KF147. The *nrpC* gene was targeted for deletion because it encodes the PPTase responsible for transferring the 4′‐phosphopantetheine moiety to the carrier protein domain of nonribosomal peptide synthetases and polyketide synthases. Removal of the PPTase prevents substrate attachment and synthesis of the NRPS/PKS product (Beld, Sonnenschein, Vickery, Noel, & Burkart, 2014). Static incubation in ATL revealed that the NRPS/PKS system did not provide a specific advantage to *L. lactis* during exponential phase growth in this medium, as both KF147 and BAL1 exhibited the same growth rates and no differences in final cell yields were observed (data not shown).

**Figure 2:**
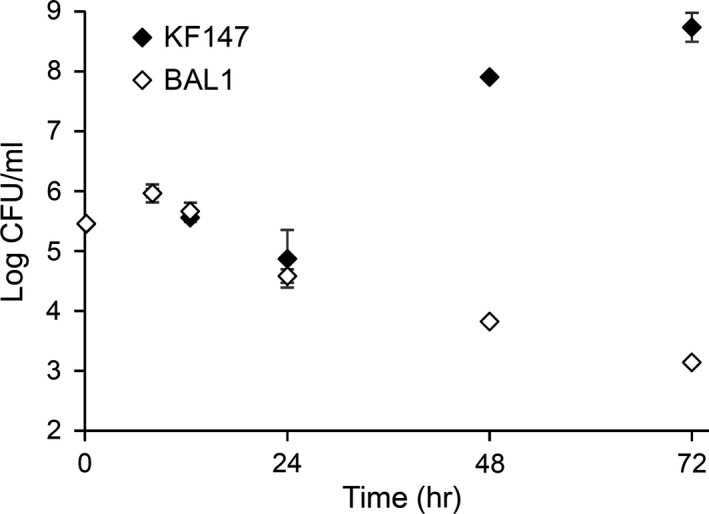
Growth of *L. lactis* KF147 and BAL1 in ATL with paraquat. *L. lactis* KF147 and BAL1 were inoculated into 10 ml ATL containing 10 mmol/L paraquat and incubated at 30°C without aeration. The average ± *SD* of three replicate cultures is shown

Because the NRPS/PKS system from *L. lactis* KF147 is homologous to a system in *S. mutans* strains UA140 and LJ23, we reasoned that the requirement for the NRPS/PKS product could depend on the extent to which reactive oxygen species are released by the plant tissues. To study the possible function of the NRPS/PKS system in tolerance of oxidative stress on plants, we supplemented ATL with 10 mmol/L paraquat, a redox cycling compound that generates superoxide radicals in the presence of reducing agents (Lock & Wilks, 2010). Under those conditions, both KF147 and the Δ*nrpC* mutant BAL1 exhibited no growth for 24 hr (Figure 2). By 72 hr, wild‐type KF147 had grown to high cell densities, whereas BAL1 viable cell numbers declined by 10‐fold (Figure 2). These results indicate that paraquat undergoes redox cycling by reducing agents in ATL and generates superoxide radicals against which the NRPS/PKS product provides protection.

### 3.4 *L. lactis* KF147 NRPS/PKS provides limited advantages during growth in standard laboratory culture medium

No differences in growth rate or final cell density were observed between *L. lactis* KF147 and BAL1 during static incubation in GM17 at 30°C, the standard laboratory culture medium and growth conditions for this species (data not shown). Moreover, unlike *S. mutans* UA140, there was no visible change in cellular pigmentation between KF147 and the Δ*nrpC* mutant.

**Figure 3:**
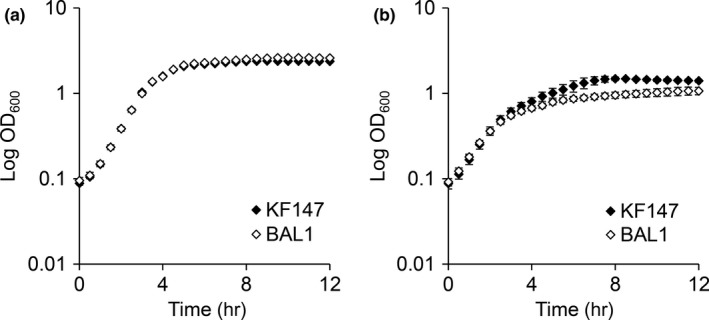
Growth of *L. lactis* KF147 and BAL1 in GM17 under respiratory conditions with and without paraquat. *L. lactis* KF147 and BAL1 were inoculated into GM17 under respiratory conditions (continuous aeration and 10 μg/ml hemin) (a) or respiratory conditions and 20 mmol/L paraquat (b). Bacteria were incubated at 30°C in microtiter plates and OD was measured at 600 nm. The average ± *SD* of at least eight replicate cultures is shown

To provide a more oxidative environment, KF147 and the BAL1 mutant were grown in the presence of paraquat. Wild‐type KF147 and the Δ*nrpC* mutant BAL1 were both unaffected by the presence of paraquat in GM17 (data not shown). In conditions conducive for *L. lactis* respiratory metabolism (*i.e*. hemin supplementation and aeration), sufficient reducing agents (e.g. NADH dehydrogenases NoxA and NoxB) might be present and could induce paraquat redox cycling and subsequent superoxide formation. Under respiratory conditions without paraquat, no change in growth was observed between KF147 and BAL1 (Figure 3a) whereas in the presence of paraquat, both strains exhibited a biphasic growth pattern (Figure 3b). During the first 2.5 hr, both strains exhibited significantly decreased growth rates (μ = 0.744 ± 0.024 hr^−1^ and μ = 0.671 ± 0.016 hr^−1^ for KF147 and BAL1, respectively) (Figure 3b) compared to exposure to respiratory conditions in the absence of this compound (*p *<* *.0005 for both) (Figure 3a). The growth impairment was greater for BAL1 than for wild‐type KF147 in the presence of paraquat (*p *<* *.0005). After 2.5 hr, the growth rates of both strains slowed considerably (μ = 0.225 ± 0.013 hr^−1^ and μ = 0.144 ± 0.008 hr^−1^ for KF147 and BAL1, respectively) and the deletion mutant was again more impaired (*p *<* *.0005) (Figure 3b). Similarly, exposure of *L. lactis* KF147 and BAL1 to the superoxide‐generating reagents xanthine oxidase (XO) and xanthine under static conditions in GM17 also resulted in a subtle but significant decrease in growth rate for BAL1 compared to KF147 (Table 2).

**Table 2:** Growth rates (hr^−1^) of *L. lactis* KF147 and BAL1 exposed to ROS

ROS	KF147	BAL1	*p*
Xanthine & XO	0.581 ± 0.036	0.535 ± 0.009	.022
H_2_O_2_	0.465 ± 0.017	0.386 ± 0.026	<.0005

The addition of H_2_O_2_ to GM17 also resulted in a significantly decreased growth rate for BAL1 as compared to KF147 (Table 2). Both *L. lactis* and *S. mutans* lack catalase (Price, Zeyniyev, Kuipers, & Kok, 2011), and therefore the NRPS/PKS in these strains appears to also provide an alternate mechanism for protection against H_2_O_2_. Overall, although the growth impairment of BAL1 was significant under oxidative conditions in GM17, the effect was not as great as in ATL. This difference might have been due to the relatively reduced expression of the NRPS/PKS gene cluster in GM17 and/or to a lack secondary metabolite precursors in the laboratory culture medium.

## 4 CONCLUSIONS

Although numerous LAB species are annotated to contain NRPS/PKS systems, few have been biochemically characterized and these are limited to *Lactobacillus reuteri* TMW1.656 (Lin et al., 2015) and *S. mutans* UA159 (Joyner et al., 2010), and UA140 (Wu et al., 2010). Remarkably, the *L. lactis* KF147 NRPS/PKS system is similar in both gene structure and function to the NRPS/PKS in *S. mutans* UA140 and LJ23 and not to orthologous systems in other lactic acid bacteria. Both of the latter species are members of the *Streptococcaceae*, however, they inhabit distinct ecological niches and it is unclear as to in which species the NRPS/PKS system originated (or perhaps a third as of yet unknown species). In addition to the NRPS/PKS system, the genetic locus also contains a gene annotated as *avrD* (llkf_1224). It is notable that this protein is also produced by certain plant pathogens and is recognized by plant host resistance (R) proteins to result in plant production of an oxidative burst and generation of superoxide radicals (Lamb & Dixon, 1997; Spoel & Dong, 2012). This finding is consistent with our results showing that the production of the NRPS/PKS appears to be dispensable for *L. lactis* during growth in standard laboratory media, but relevant to the plant environment, possibly either to counter responses to pathogenic bacteria (oxidative burst) or to survive generally, aerobic conditions on leaf tissues. In conclusion, tolerance to ROS compounds due to expression of an NRPS/PKS product represents a novel strategy for *L. lactis* ROS detoxification, possibly during growth on living plants, and with potential application to support the viability of this organism in culture production and preservation.

## CONFLICT OF INTEREST

None declared.
