# Supplementary material for: The Lactococcus lactis KF147 nonribosomal peptide synthetase/polyketide synthase system confers resistance to oxidative stress during growth on plant leaf tissue lysate
Source: Microbiologyopen. 2017 Sep 18;7(1):e00531. doi: 10.1002/mbo3.531 (PMC5822349; doi:10.1002/mbo3.531)
Supplement: Supplementary file 1 [file MBO3-7-na-s001.docx]

TABLE S1. Primers used in this study.

ID	Primer	Sequence (5' - 3')[1,2]	Description
**A**	nrpC_up_for_SalI	GCGAAAGTCGACTAATTTGTTTGCCTTTTGCGGG	Upstream flanking region of *nrpC*
**B**	nrpC_up_rev_linker	**GAAGCAGCTCCAGCCTACAC**TGTTTCCATCCATCTATATCTT	Upstream flanking region of *nrpC*
**C**	nrpC_down_for_linker	**GTGTAGGCTGGAGCTGCTTC**AAAGAGACAAGAAATAAAGGATGTA	Downstream flanking region of *nrpC*
**D**	nrpC_down_rev_SacII	GCGAAACCGCGGAGCACTCATTACAAATTGTTCA	Downstream flanking region of *nrpC*
**E**	nrpA_qPCR_for	CCTGAGCCAAATGGGTTATC	Internal region of *nrpA* for qPCR
**F**	nrpA_qPCR_rev	TGTCCAAGATGGGGAAATTG	Internal region of *nrpA* for qPCR
**G**	nrpC1_qPCR_for	TGACCTGCGGCATTTTACTCA	Internal region of *nrpC* for qPCR
**H**	nrpC1_qPCR_rev	CGAACGTTTCTTATCTGCATATCGT	Internal region of nrpC for qPCR

[1]Underlined sequence indicates restriction enzyme cut site.

[2]Bolded letters indicates 5' reverse complementary linker sequences
